# Test-retest reliability of a smartphone-based approach-avoidance task: Effects of retest period, stimulus type, and demographics

**DOI:** 10.3758/s13428-022-01920-6

**Published:** 2022-08-01

**Authors:** Hilmar G. Zech, Philip Gable, Wilco W. van Dijk, Lotte F. van Dillen

**Affiliations:** 1 Leiden University, Leiden, The Netherlands; 2 Technical University Dresden, Dresden, Germany; 3 University of Delaware, Newark, DE USA; 4 Knowledge Centre Psychology and Economic Behaviour, Leiden, The Netherlands

**Keywords:** Approach-avoidance, Reliability, Test-retest, Split-half, AAT, Reaction time, Force

## Abstract

The approach-avoidance task (AAT) is an implicit task that measures people’s behavioral tendencies to approach or avoid stimuli in the environment. In recent years, it has been used successfully to help explain a variety of health problems (e.g., addictions and phobias). Unfortunately, more recent AAT studies have failed to replicate earlier promising findings. One explanation for these replication failures could be that the AAT does not reliably measure approach-avoidance tendencies. Here, we first review existing literature on the reliability of various versions of the AAT. Next, we examine the AAT’s reliability in a large and diverse sample (*N* = 1077; 248 of whom completed all sessions). Using a smartphone-based, mobile AAT, we measured participants’ approach-avoidance tendencies eight times over a period of seven months (one measurement per month) in two distinct stimulus sets (happy/sad expressions and disgusting/neutral stimuli). The mobile AAT’s split-half reliability was adequate for face stimuli (*r* = .85), but low for disgust stimuli (*r* = .72). Its test–retest reliability based on a single measurement was poor for either stimulus set (all ICC1s < .3). Its test–retest reliability based on the average of all eight measurements was moderately good for face stimuli (ICCk = .73), but low for disgust stimuli (ICCk = .5). Results suggest that single-measurement AATs could be influenced by unexplained temporal fluctuations of approach-avoidance tendencies. These fluctuations could be examined in future studies. Until then, this work suggests that future research using the AAT should rely on multiple rather than single measurements.

The approach-avoidance task (AAT) is an implicit task that measures people’s behavioral tendencies to approach or avoid stimuli in their environment (Solarz, 1960). For example, most people tend to approach happy faces but avoid angry faces (Rotteveel & Phaf, 2004). Such tendencies are often adaptive and can even be crucial for survival (Schneirla, 1959). Biased approach-avoidance tendencies, on the other hand, could explain why some people suffer from health problems and others do not (Hofmann et al., 2008). Consequently, several researchers have attempted to link individual differences in approach-avoidance tendencies to individual differences in health-related variables. This effort has already been fruitful: Biased approach tendencies have been reported in drinkers towards alcohol, in sm okers towards cigarettes, and in people with a high body mass index (BMI) towards food (Ernst et al., 2014; Maas et al., 2017; Wiers et al., 2013). Likewise, biased avoidance tendencies have been reported in clinical groups, such as in arachnophobe away from spiders and in socially anxious people away from emotional faces (Rinck & Becker, 2007; Roelofs et al., 2010). In these studies, individual differences in approach-avoidance tendencies can explain individual differences in (mental) health-related variables.

Given these promising findings, it is unfortunate that many of the above effects have failed to be consistently replicated (for overviews, see Kakoschke et al., 2019 and Loijen et al., 2020). For example, using the same methods as Ernst et al. (2014), Wiers et al. (2017) did not find stronger alcohol approach tendencies in alcohol-dependent compared to healthy participants. Machulska et al. (2015) did not replicate Wiers et al.’s (2013) findings that smokers had stronger approach tendencies to cigarettes than nonsmokers. In Maas et al.’s (2017) study, participants with higher BMIs displayed stronger food approach tendencies towards sweet but not towards salty foods. Other researchers, moreover, found no relationship between participants’ BMIs and their food approach tendencies (Kakoschke et al., 2015, 2017a, 2017b; Schumacher et al., 2016). Given the social importance of approach-avoidance tendencies in health problems and the potential of the AAT to help explain why some people suffer from them, it is crucial to understand why the AAT results in some studies link biased approach-avoidance tendencies to individual differences in health outcomes and others do not^1^.

One explanation for these inconsistent findings could be that the AAT does not reliably measure approach-avoidance tendencies. Reliability refers to the temporal stability with which a task can measure a construct (Kaplan & Saccuzzo, 2017). It can be measured both within one measurement session (split-half reliability) and across several sessions (test-retest reliability). Test-retest reliability is important when aiming to replicate effects, because low test-retest reliability limits the correlation that can be observed between two variables (Spearman, 1904/2010). For example, if the actual correlation between food approach tendencies and BMI was *r =* .40, but the retest reliability of the AAT was only .20, the observed correlation would fall to *r =* .18 (the correlation observed by Schumacher et al., 2016). This decreased correlation would then decrease power and ultimately could explain failed replications.

Other behavioral tasks—such as the stop-signal task and the go/no-go-task—have already been shown to be unreliable: After examining 374 measures from various tasks (total *N* = 17,550), Enkavi et al. (2019) concluded that “most individual dependent measures from [implicit] tasks are not appropriate for individual difference analyses based on their low [test-retest] reliability.” Consequently, several authors have suggested that replication failures in AAT research could also be explained by the task’s (assumed) low reliability (Aupperle et al., 2011; Becker et al., 2019; Field et al., 2016; Gawronski et al., 2011; Kakoschke et al., 2015; Loijen et al., 2020; Meule, Richard, et al., 2019b; Reddy et al., 2016; Reinecke et al., 2012; Struijs et al., 2017, 2018; Swinkels et al., 2019; Voncken et al., 2012; Vrijsen et al., 2018; Wiers et al., 2013; Zech et al., 2020).

However, reports of the AAT’s test-retest reliability are scarce. In a systematic review (for details see Appendix B; Fig. 7), we identified 205 studies that used different versions of the AAT^2^. Of these only four reported on the task’s test-retest reliability and all of these studies reported reliabilities too low for testing individual differences (*rs* < .36; see Table 1). Moreover, these reports do not follow current recommendations for assessing and reporting test-retest reliability (Mokkink et al., 2010; Oosterwijk et al., 2019; Polit, 2014). Specifically, sample sizes in these studies were relatively small (*Ns* < 150) and they do not report confidence intervals around their estimates. This makes it likely that these studies over- or underestimated the task’s true reliability (Giraudeau & Mary, 2001; Oosterwijk et al., 2019; Polit, 2014). Further, the homogeneity of their samples (e.g., young university students; alcohol-dependent participants), the focus on single stimulus types (e.g., spiders), and the retest periods make it difficult to generalize their findings to other studies. Past study designs were limited in that they did not allow testing the influence of stimulus type, retest-period, and demographics on reliability. Moreover, since the publication of these studies, improved ways of conducting the AAT and calculating approach-avoidance tendencies have been established (Kersbergen et al., 2015; Lender et al., 2018; Meule, Richard, et al., 2019b; Phaf et al., 2014; Rotteveel & Phaf, 2004; Zech et al., 2020). Together, these recent advancements might increase the task’s reliability.

**Table 1:** Overview of studies reporting test-retest reliability

Study	Sample (analyzed)	Task(s)	Stimuli	Retest period	Retest reliability
Kahveci et al. (2020)	60 female students	Swiping AAT (relevant feature)	Foods vs. objects	1 week	*r =* .23
Piercy et al. (2021)	117 participants with alcohol use disorder	Joystick AAT (irrelevant feature)	Alcohol vs. non-alcohol	4 days	*r =* .027
Reinecke et al. (2010)	75 students	Joystick AAT (irrelevant feature)	Spiders vs. butterflies	3 to 21 days (mean: 9 days)	*r* = .35
Rinck et al. (2018)	143 abstinent alcohol-dependent inpatients	Joystick AAT (irrelevant feature)	Alcohol vs. non-alcohol	1 year	*r* = .01

This table gives an overview of studies reporting on the AATs test-retest reliability. The mean test-retest reliability reported by these studies was .15

Additionally, for the past decade, researchers have recommended calculating reliability based on intraclass correlation coefficients (ICCs) rather than based on test-retest (Pearson) correlations (Mokkink et al., 2010; Polit, 2014; Qin et al., 2019). The advantage of using ICCs over simple correlations is that they allow for more general tests of a task’s reliability (Bartko, 1966; Koo & Li, 2016; McGraw & Wong, 1996; Shrout & Fleiss, 1979). In general, (conceptually similar to Pearson correlations) ICCs can be understood as the ratio between wanted variance and total variance (wanted + unwanted variance). Unlike correlations, ICCs allow to distinguish between cases in which between-session variance is wanted (e.g., intervention studies or studies focusing on states) and cases in which between-session variance is unwanted (e.g., cross-sectional studies focusing on traits). In the latter case, several measurement sessions of a task could be completed—not because researchers are interested in session differences, but simply to obtain a more reliable (average) participant score.

The current study aims at providing researchers with a more conclusive, generalizable, and up-to-date estimate of the AAT’s (test-retest) reliability. We sought to overcome many of the weaknesses of past studies measuring the reliability of the AAT. Specifically, we assessed the AAT’s reliability in a large and diverse sample of 1077 participants (248 of whom completed all eight sessions). This large sample allowed us to give precise estimates of the task’s reliability and report narrow confidence intervals. To assess the generalizability of our findings, we also tested whether reliability differs in different subsamples (men, women, old, and young participants). We further assessed the task’s reliability based on two stimulus sets: emotional expressions and pictures of disgusting scenes, to explore whether reliability depends on the stimuli used in the AAT. To potentially increase reliability, we used an updated, relevant-feature version of the AAT, which decreases measurement error by focusing participants’ attention on the stimulus dimension of interest (e.g., the emotional expression of faces; Phaf et al., 2014). We also report reliabilities based on a novel way of calculating approach-avoidance tendencies based on mixed models (Zech et al., 2020). It has recently been suggested that mixed models could give a more precise estimation of a task’s reliability, as unlike traditionally used aggregation methods, they do not compound measurement error and systematic variance (Haines et al., 2020)^3^. Finally, we tested participants eight times with retest intervals of one month. The increased number of measurements should increase sensitivity and also allow us to examine whether repeating tests can make the AAT more reliable. It also should allow us to explore the presence of training effects and to understand how test-retest reliability changes at different test–retest periods (one to seven months). Finally, the long test–retest period should decrease the potential influence of carryover effects that could overestimate the task’s test-retest reliability. This improvement is especially important for researchers who aim to correlate approach-avoidance tendencies with slow-changing individual difference variables such as addiction status, phobias, or BMI.

Testing participants over an extended period of time would have been difficult using traditional versions of the AAT that rely on stationary lab-based setups. To overcome this problem, recently, several mobile versions of the AAT have been developed (e.g., Meule, Richard, et al., 2019b; Zech et al., 2020). These mobile AATs have the advantage over classical versions of the task because they run entirely on smartphones and can be easily deployed in field research and in longitudinal studies. This advantage makes them uniquely suited to study how approach-avoidance tendencies are influenced by interventions (such as interventions to reduce alcohol consumption, e.g., Wiers et al., 2013) and—more importantly for the current purposes—how stable approach-avoidance tendencies are over time in the absence of interventions. The validity of one of these tasks was already established in a recent study (Zech et al., 2020). Yet, its test-retest reliability has, so far, not been assessed. As temporal stability is an important precondition to determine the relations with individual differences and the effects of interventions, and as mobile AATs are especially suited to study such dynamics, it is especially important to assess its reliability.

In addition, mobile versions of the AAT can be used to better understand why approach-avoidance tendencies fluctuate. A task’s low test-retest reliability does not necessarily mean that the task is not a useful measurement. As Enkavi et al. (2019) note, low test-retest reliability is only problematic if a task is used as a trait-measure^4^—in which case, low test-retest reliability is a sign of large measurement error. When used as a state measure, on the other hand, temporal changes underlying low test-retest reliability are less problematic and can even be desirable. In this case, rather than test-retest reliability, split-half reliability becomes the more important psychometric criterion, as it determines the stability with which a task measures a construct within one measurement session (Hedge et al., 2018). Several studies have already assessed the split-half reliability of laboratory-based AATs, but estimates vary greatly (*rs* ranging from −.24 to .97; *M =* .52; *SD* = .30; see Appendix C; Table 3). To give an indication whether approach-avoidance tendencies are more trait- or more state-like, we therefore also assessed the AAT’s split-half reliability.

Understanding the AAT’s reliability is crucial for researchers who use the task to understand individual differences, as well as researchers using the task to assess the efficacy of interventions. A possible lack of temporal stability, especially in the presence of low split-half reliability, might indicate that approach-avoidance tendencies are less stable than current theories suggest and could open the door to new research which, instead of focusing on how approach-avoidance tendencies differ between individuals, could examine how our approach-avoidance tendencies change with time and context. In either case, a better understanding of the AAT’s reliability provides a crucial first step.

## Method

### Participants

**Fig. 1:**
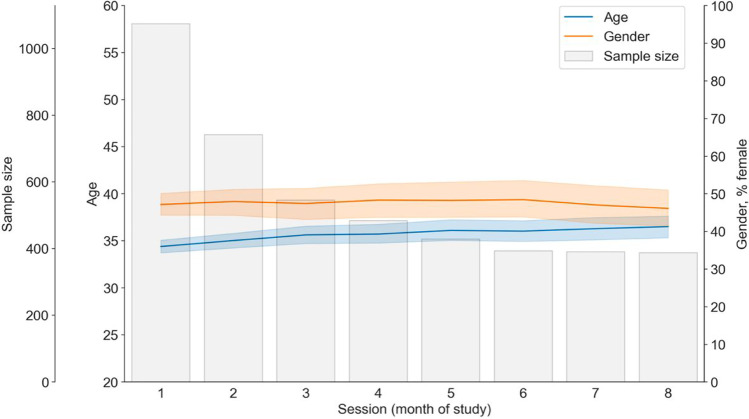
Attrition and change in sample characteristics over study sessions. This figure shows the change of sample characteristics over time. The *x*-axis shows months since beginning of study. Grey bars show the sample size for each session. The blue line shows the change in the sample's mean age (corrected for within-participant age changes) and the orange line shows the change in percentage female

Participants were unselected US citizens recruited from various regions via the online recruitment platform Prolific (www.prolific.co). Whereas 1077 participants completed one session one, 248 participants completed all eight sessions (see Fig. 1). Participants' ages ranged from 18 to 76 years (*M* = 34.91, *SD* = 11.29); 561 (50.3%) reported being male, 526 (47.1%) identified as female, and 29 (2.6%) as nonbinary/third gender. Regression analyses indicated that the average sample age increased slightly across sessions (*b* = .39 [years per consecutive session], *t* = 5.46, *p* = < .001). The gender distribution, however, did not change across sessions (*p*s > .6 see, Fig. 1 and analyses on the project’s Open Science Framework page (https://osf.io/t3f4y/).

## Procedure

As part of a larger survey investigating experiences during the COVID-19 pandemic, participants completed the AAT. After signing up on Prolific Academic, participants downloaded the study app on which the rest of the study was completed. Next, they completed eight measurement sessions over a period of seven months (one month between each session). In each of the sessions, they first completed the informed consent and filled in their unique Prolific ID. Next, they answered basic demographic questions (age, gender) and filled in several COVID-19-related questionnaires for the overarching project. Included in these questionnaires was the pathogen subscale of the Three Domain Disgust Scale (Tybur, 2009) and several questions about their emotional state which will be used for the current project. After filling in the questionnaires, participants completed two AATs—one with pictures of emotional expressions (emotional expressions AAT) and one with pictures of disgusting scenes (disgust AAT; see below for details). After each session, they were compensated for their participation.

## Materials

### Mobile AAT

Participants completed two mobile AATs on their own smartphones (emotional expressions AAT and disgust AAT). During each AAT, they were presented with pictures on their phone’s screen and responded to the pictures by pulling the phone toward themselves (approach) or pushing the phone away from themselves (avoidance). Each AAT consisted of two blocks—one, in which participants had to approach one stimulus type (e.g., happy faces) and avoid the other (e.g., sad faces), and one block in which these instructions were reversed (e.g., push away happy faces and pull sad faces). Each block consisted of 40 trials, with a break after 20 trials. Each block was preceded by a practice block (8 trials) in which participants received accuracy feedback for their responses. During each response, the phone’s movement sensors detected the phone’s acceleration, from which reaction times (RTs) were later calculated. These RTs were then used to calculate approach-avoidance tendencies.

### Stimulus sets

#### Emotional faces

The emotional faces consisted of 20 happy and 20 sad faces, taken from the FaceGen Modeller v3.5 (Singular Inversions; Roesch et al., 2011). Half of the faces were male and half were female. Half the ethnicities were Caucasian and half were East Asian.

#### Disgusting stimuli

The disgusting stimuli consisted of 20 disgusting and 20 neutral stimuli, taken from the Culpepper Disgust Image Set (Culpepper et al., 2018). Disgusting stimuli depicted highly disgusting scenes such as vomit on the ground and neutral stimuli were visually comparable but lacked the disgust trigger (e.g., same ground without vomit).

## Analysis

### Data preprocessing

We followed the preprocessing procedure outlined in Zech et al. (2020). After extracting reaction times (RTs) and movement direction from raw acceleration data, we removed practice trials, error trials, trials with missing sensor data, trials with implausibly short reaction times (< 200 ms), and trials with low absolute maximum forces (< 1 m/s^2^; indicating nonresponses; in total 9% of experimental trials in the disgust AAT and 7.5% of experimental trials in the facial expressions AAT removed). Stimuli with overall error rates higher than 20% (5 stimuli; 5.8% of the data; all removed stimuli came from the disgust AAT) and data of participants with fewer than 70% valid experimental trials (101 participants; 8.6% of initial 1172) were also removed. Note that in the remaining sample the average error rate was low (< 10%; see supplementary materials). Data preprocessing was performed using Python (version 3.5.5). All preprocessing scripts and the complete data (including excluded trials/participants) are available on the project’s Open Science Framework page (https://osf.io/t3f4y/).

### Modeling

Traditionally, approach-avoidance tendencies are calculated using double-difference scores of median RTs (e.g., Rotteveel & Phaf, 2004). For example, to calculate the approach tendency for happy versus sad faces, most researchers would first calculate median RTs for each trial category and then apply the following formula to calculate approach-avoidance tendencies:

- *Happy approach tendency = (push_happy – pull_happy) – (push_sad – pull_sad)*

As suggested by Zech et al. (2020), here, we instead used linear mixed-effects models (LMMs) to calculate participants’ approach-avoidance tendencies. Approach-avoidance tendencies were modeled as the interaction effect between response direction (*is_pull*) and stimulus type (*is_happy* or *is_disgusting*) with inverted RTs (*1/RT*) as the outcome variable^5^. To estimate participants’ approach-avoidance tendencies separately for each session, interacting random effects for participant and session were also modeled (*session : pp*). To test for time effects, we also added session number (*session_number)* to the model as a fixed effect. The main regression models for the emotional expressions and for the disgust AAT were therefore defined as (for notation see Bates, 2005):

- 1/RT ~ is_pull * is_happy * session_number + (is_pull * is_happy | session : pp)

- and
- 1/RT ~ is_pull * is_disgusting * session_number + (is_pull * is_disgusting | session : pp)

### Validity and temporal fluctuations

To examine the tasks’ validity, we assessed the two-way interactions between response direction (*is_pull*) and stimulus type (*is_happy* and *is_disgusting* in the emotional expressions AAT and the disgust AAT, respectively). We expected this interaction to be positive in the emotional expressions AAT, indicating an approach tendency for happy compared to sad faces, and negative in the disgust AAT, representing an avoidance tendency away from disgusting compared to neutral stimuli. To examine time effects, we assessed the three-way interactions between response direction, stimulus type, and session number.

### Test-retest reliability

To calculate test-retest reliabilities, we first extracted random slopes from the above model for each session of each participant. These random slopes indicate how much each participant’s approach-avoidance tendency in each session deviated from the average approach-avoidance effect in the study. We then calculated ICCs based on these random slopes using the ICC function from the R psych package (version 2.0.12; Revelle, 2019). This function has the advantage of calculating ICCs based on mixed models, which can include participants with missing sessions into the calculation. Next, as suggested by Liljequist et al. (2019), we chose the adequate ICC by comparing unbiased ICCs, consistency ICCs, and absolute agreement ICCs. In theory these three ICCs can differ if sessions differ systematically (Shrout & Fleiss, 1979). However, as in our data the values from these three ICCs were very similar (see online materials) and so we only report unbiased ICCs (ICC1s, as suggested by Liljequist et al., 2019).

In addition to single-measure ICCs (ICC1), we also calculated average-measure ICCs (ICC1ks)^6^. ICC1ks represent the task’s test-retest reliability, when scores are not based on a single measurement, but on several measurements (eight in our study; Shrout & Fleiss, 1979). To get insight into how participant variables affect ICCs, we also calculated ICCs separately for men and women and for younger (below the median age of 33 years) and older (above the median age) participants. To understand how test-retest reliabilities change with increasing retest periods, we also calculated the effect of the retest-period length on test-retest reliability. Finally, as the test-retest reliability of difference scores is usually lower than that of their compounds, we also report the test-retest reliability of mean reaction times (on which approach-avoidance tendencies are based).

For each ICC, we calculated 95% confidence intervals using the psych package, which implements the method outlined by Shrout and Fleiss (1979). Different ICCs were compared based on the overlap of confidence intervals. Qualitative interpretations were given in accordance with Koo and Li (2016) based on confidence intervals. ICCs less than .5 were interpreted as “poor,” ICCs between .5 and .75 as “moderate,” ICCs between .75 and .9 as “good,” and ICCs above .9 as “excellent.” For confidence intervals that included two of these cutoffs, both interpretations were given (e.g., “moderate to good”).

### Split-half reliability

Split-half reliability was estimated for each session separately. To do so, we first split the data from each session into two datasets (based on even and odd trial numbers). We next fitted separate models for each of these splits, extracted the per participant random slopes for approach-avoidance effects, and correlated the resulting random slopes against each other. Finally, we applied the Spearman-Brown correction to account for the halved number of trials in each model.

To give qualitative interpretations to the split-half reliabilities, we followed suggestions by Nunnally and Bernstein (1994)^7^. Split-half reliabilities between .8 and .9 were labeled as adequate for basic research, split-half reliabilities between .9 and .95 as adequate when important decisions are made about individuals (e.g., when treated as a diagnostic criterium for treatment decisions).

## Results

### General approach-avoidance effects (validity and time effects)

Before assessing the AAT’s reliability, we first validated the task by testing the expected overall approach-avoidance effects across all sessions. We expected that participants, in general, would have an approach tendency towards happy compared to sad faces and an avoidance tendency away from disgusting compared to neutral stimuli. We also assessed time effects, as the interaction between session number and approach-avoidance effects.

#### Emotional faces

**Fig. 2:**
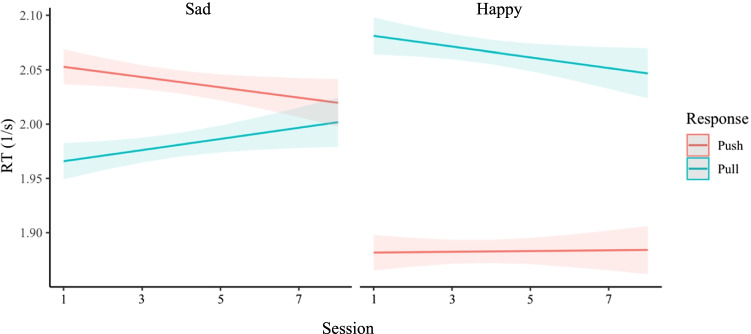
Approach tendencies to happy compared to sad faces. Note. These plots show inverted reaction times (y-axis; note that higher is faster) over session number (x-axis) for the emotional faces stimuli AAT (Sad, Happy). The colors represent responses (red: push, green: pull). The left panel shows responses to sad stimuli and the right panel responses to happy stimuli. It can be seen that, in general, participants have an approach tendency to happy faces, as they are faster to approach compared to avoiding happy faces but faster to avoid compared to approaching sad faces. It can also be seen that this effect decreases in later sessions

Modeling the data from the emotional expressions AAT revealed main effects of response direction and stimulus type. In general, participants reacted slower towards happy compared to sad faces (*b* = −0.028 [−0.033, −0.021], *t* = −9.86, *p* = < .001) and faster when pulling compared to pushing stimuli (*b* = 0.056 [0.051, 0.066], *t* = 16.50, *p* = < .001). Importantly, there was a significant two-way interaction between response direction and stimulus type (*b* = 0.286 [0.260, 0.310], *t* = 26.04, *p* = < .001), revealing the expected happy approach tendency (see Fig. 2). There was also a significant three-way interaction between response direction, stimulus type, and session number (*b* = −0.015 [−0.022, −0.010], *t* = −4.95, *p* = < .001), indicating that approach-avoidance effects decreased over time (see Fig. 2).

#### Disgusting stimuli

**Fig. 3:**
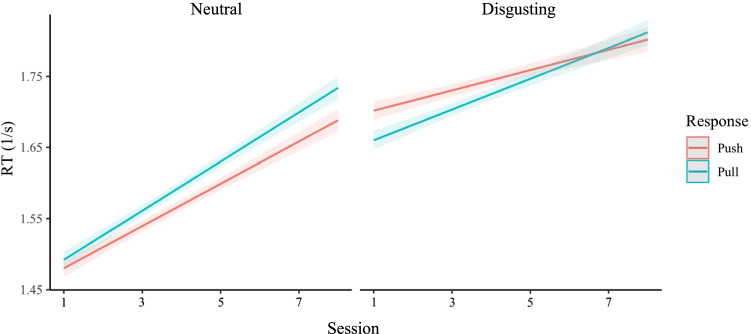
Avoidance tendencies away from disgusting compared to neutral stimuli. Note. These plots show inverted reaction times (y-axis) over session number (x-axis) for the disgusting stimuli AAT. The colors represent responses (red: push, green: pull). The left panel shows responses to neutral stimuli and the right panel responses to disgusting stimuli. It can be seen that, in general, participants have an avoidance tendency away from disgusting stimuli, as they are slower to approach compared to avoiding disgusting stimuli but faster to approach compared to avoiding neutral stimuli. It can also be seen that average inverted reaction times increase in later sessions

Modeling the data from the disgust AAT revealed main effects of response direction and stimulus type. In general, participants reacted faster towards disgusting compared to neutral objects (*b* = 0.195 [0.186, 0.203], *t* = 58.33, *p* = < .001) and slower when pulling compared to pushing stimuli (*b* = −0.015 [−0.021, −0.009], *t* = −5.57, *p* = < .001). Importantly, there was a significant two-way interaction between response direction and stimulus type (*b* = −0.054 [−0.068, −0.036], *t* = −7.20, *p* = < .001), indicating that participants, on average, had an avoidance tendency away from disgusting (compared to neutral) objects (see Fig. 3). We, therefore, concluded that the AAT successfully measured the expected approach-avoidance tendencies for disgusting and neutral stimuli (see Fig. 3). Finally, there was no significant three-way interaction between response direction, stimulus type, and session number (*b* = 0.003 [−0.001, 0.007], *t* = 1.24, *p* = .214), indicating that general approach-avoidance effects did not change over time (see Fig. 3).

### Test-retest reliability

#### Emotional faces

**Fig. 4:**
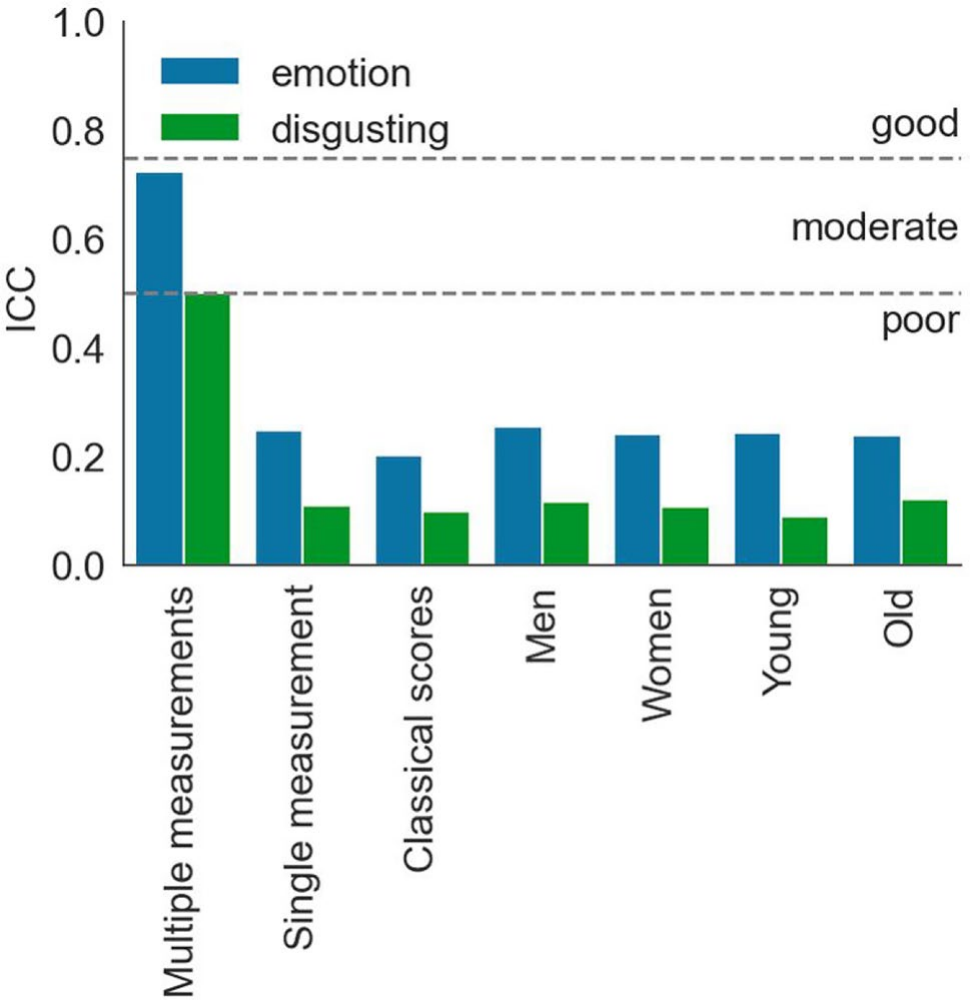
Test-retest reliabilities by task, type, and subsample. This figure summarizes ICCs (*y*-axis) by task (color), type, and subsample (*x*-axis). Striped lines indicate qualitative interpretations of ICCs. It can be seen that only ICCs based on several measurements (ICCk1s) reached moderate to good reliability. Test-retest reliabilities of the emotional expressions AAT were consistently higher than those of the disgust AAT—a point we further address in the discussion. Sample characteristics, such as age and gender, had no effects on ICCs

**Fig. 5:**
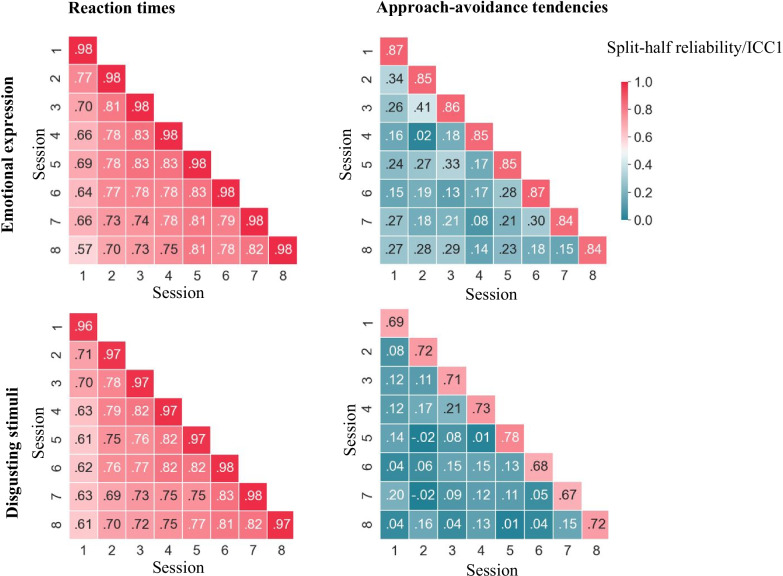
Split-half and test-retest reliabilities per session and combination of sessions. This figure shows split-half reliabilities per session (diagonals) and test-retest reliability (ICC1s) for all session combinations (off diagonals). Upper panels show data from the emotional expressions AAT and lower panels from the disgust AAT. Left panels show statistics for reaction times and right panels for approach-avoidance tendencies. It can be seen that both split-half and test-retest reliabilities for reaction times are high. For approach-avoidance tendencies, however, only split-half reliabilities are high, whereas test-retest reliabilities are low. Reliabilities are generally lower for the disgust AAT compared to the emotional expressions AAT. For reaction times, it can also be seen that test-retest reliabilities decrease with increasing retest periods (distance from diagonals)

Test-retest reliabilities for mean reaction times towards emotional faces were moderate to good (ICC1 *=* .75 [.74, .77]). However, test-retest reliabilities for main effects of response (ICC1 *=* .36 [.34, .38]) and stimulus type (ICC1 *=* .20 [.18, .22]) were poor. Most importantly, test-retest reliability of approach-avoidance tendencies was also poor (ICC1 *=* .25 [.23, .27]; somewhat lower when calculated based on traditional double difference scores: ICC1 = .20 [.18, .22]; and both somewhat higher than average test-retest reliabilities reported in the literature, mean *r =* .15). Test-retest reliabilities did not differ between different subsamples (based on overlapping confidence intervals; women: ICC1 = .24 [.21, .27], men: ICC1 = .26 [.23, .28], young participants: ICC1 = .24 [.22, .27], old participants: ICC1 = .24 [.21, .27]). Importantly, average-measurement ICCs (ICC1ks) were significantly higher than single-measurement ICCs, indicating that the approach-avoidance task’s test-retest reliability can be brought to moderate to good levels when scores are based on the average of multiple measurements (ICC1k = .73 [.70, .75]; for an overview of all test-retest reliabilities see Figs. 4 and 5).

#### Disgusting stimuli

Test-retest reliabilities for mean reaction times in the disgust AAT were moderate (ICC1 *=* .69 [.67, .71]). Test-retest reliabilities for main effects of response (ICC1 *=* .18 [.16, .20]) and stimulus type (ICC1 *=* .46 [.44, .48]) were poor. Most importantly, the test-retest reliability of approach-avoidance tendencies was also poor (ICC1 *=* .11 [.10, .13]; similar when calculated based on traditional double difference scores: ICC1 = .10 [.09, .11]; and both somewhat lower than average test-retest reliabilities reported in the literature, mean *r =* .15). Reliabilities did not differ between different subsamples (based on overlapping confidence intervals; women: ICC1 = .11 [.09, .13], men: ICC1 = .12 [.10, .14], young participants: ICC1 = .09 [.07, .11], old participants: ICC1 = .12 [.10, .14]). Taking multiple measurements into account did significantly increase test-retest reliability for approach-avoidance tendencies from poor to moderate levels (ICC1k = .50 [.46, .54]; for an overview of all test-retest reliabilities see Figs. 4 and 5).

#### Effect of retest period

**Fig. 6:**
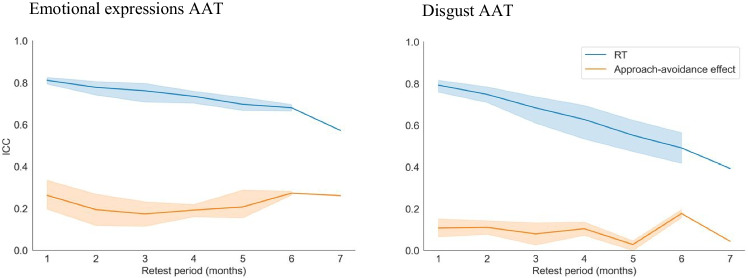
Effect of retest period on test-retest reliabilities. This figure shows the relationship between the length of the retest period (*x*-axis) and ICCs (*y*-axis) for reaction times (blue lines) and approach-avoidance tendencies (red lines). The left panel shows the relationship for the emotional expressions AAT and the right panel for the disgust AAT. It can be seen that while the reliability for reaction times decreases with increasing retest periods, the reliability of approach-avoidance tendencies remains stable. Shaded areas indicate 95% confidence intervals

For mean reaction times, there was a negative relationship between the length of the retest period and test-retest reliabilities both in the emotional expressions AAT (*b =* −0.03; i.e., reliability decreased by 3% with each session) and in the disgust AAT (*b =* −0.03; see Figs. 5 and 6). Test-retest reliabilities of approach-avoidance tendencies did not depend on the length of the test-retest interval and remained low across all intervals (*bs* < 0.001; see Figs. 5 and 6).

### Split-half reliability

#### Emotional faces

Spearman-Brown split-half reliabilities of mean reaction times indicated high reliabilities for all sessions (*M*_*r*_ *=* 0.98, *SD*_*r*_ *=* 0.00; see Table 1). Split-half reliabilities for main effects of response (*M*_*r*_ *=* 0.63, *SD*_*r*_ *=* 0.07) and stimulus type (*M*_*r*_ *=* 0.44, *SD*_*r*_ *=* 0.20) were, however, low. Most importantly, split-half reliabilities for approach-avoidance tendencies were high enough for basic research, although not high enough for making diagnostic decisions about individuals (*M*_*r*_ *=* 0.85, *SD*_*r*_ *=* 0.01). Split-half reliabilities were higher than average split-half reliabilities reported in the literature (mean *r =* .52; see Appendix C; Table 3).

#### Disgusting stimuli

Spearman-Brown split-half reliabilities of mean reaction times indicated high reliabilities for all sessions (*M*_*r*_ *=* 0.97, *SD*_*r*_ *=* 0.00; see Table 1). Split-half reliabilities for main effects of response (*M*_*r*_ *=* 0.50, *SD*_*r*_ *=* 0.24) and stimulus type (M = 0.68, *SD*_*r*_ *=* 0.07) were, however, low. Most importantly, split-half reliabilities for approach-avoidance tendencies to disgust versus neutral stimuli were too low even for basic research (*M*_*r*_ *=* 0.71, *SD*_*r*_ *=* 0.03). Split-half reliabilities were higher than average split-half reliabilities reported in the literature (mean *r =* .52; see Appendix C; Table 3).

## Discussion

The approach-avoidance task (AAT) measures people’s implicit tendencies to approach or avoid stimuli. Given its promise in explaining unhealthy or dysfunctional behaviors, a growing number of researchers have started using the approach-avoidance task as a measure of individual differences. Although test-retest reliability is an important prerequisite for using a task for this purpose, the AAT’s test-retest reliability has not yet been firmly established. To provide researchers with a more generalizable assessment of the AAT’s test-retest reliability, we assessed its reliability based on a large sample (*N* = 1077; 248 of whom completed all eight sessions), two distinct stimulus sets, an improved task design, an improved method of calculating approach-avoidance tendencies, and using a long retest period of seven months for eight consecutive monthly assessments. To achieve this long period of testing, we used a new, mobile version of the AAT that can be easily deployed in field research.

### Summary of results

We successfully validated the AAT, as it revealed both the expected general approach tendency to happy compared to sad faces and the expected avoidance tendency away from disgusting compared to neutral stimuli. In the emotional expressions AAT, this approach tendency decreased over time, whereas it remained stable in the disgust AAT. The split-half reliability of the emotional expressions AAT was adequate for basic research but too low should the AAT be used for important decisions about individuals (e.g., diagnostics; based on standards suggested by Nunnally & Bernstein, 1994). For the disgust AAT, split-half reliability was too low for either purpose. When relying on single measurements, the test-retest reliability for both the emotional expressions AAT and the disgust AAT was too low for using it to test individual differences (“poor” based on standards by Koo & Li, 2016). However, when relying on all eight measurements, the test-retest reliability of the emotional expressions AAT increased to moderate to good and the test-retest reliability of the disgust AAT increased to moderate. For both tasks, the test-retest reliabilities were somewhat lower when calculating approach-avoidance tendencies based on traditional double difference scores compared with scores derived from mixed models. Neither task’s test-retest reliability depended on basic participant characteristics (age, gender) or the length of the retest period.

### Limitations

In this study the smallest retest period was one month. A task’s test-retest reliability depends heavily on the length of this retest period (Kaplan & Saccuzzo; Polit, 2014). Therefore, to adequately assess a task’s test-retest reliability requires knowledge of the stability of the underlying processes the task aims to measure (Polit, 2014). Should underlying processes change more rapidly than the retest period, these changes can explain poor test-retest reliability. In other words, the poor test-retest reliability found in the current study might be a consequence of the study design, rather than the task’s inability to reliably measure approach-avoidance tendencies. To our knowledge it is not known at which frequency different approach-avoidance tendencies change as existing studies have mostly focused on cross-sectional designs. It is therefore possible that the targeted approach-avoidance tendencies changed at a higher frequency than the frequency of our retests. An indication for this comes from the absence of an effect of retest-period length on reliability. In general, test-retest reliability should decrease with increasing retest-periods (Hedge et al., 2018). It is possible that in this study the task’s test-retest reliability already reached its minimum before the end of our first retest period. Future studies should therefore assess the AAT’s reliability at shorter time intervals to determine the frequency at which different approach-avoidance tendencies fluctuate. It should however be noted that the currently used long retest period is relevant for researchers who use the AAT to study slow-changing variables, such as addiction status (e.g., Wiers et al., 2013), phobias (e.g., Rinck & Becker, 2007), or BMI (Havermans et al., 2011).

Our finding that test-retest reliability was low while split-half reliability (at least for the emotional expressions AAT) was high indicates that approach-avoidance tendencies fluctuate with time (Hedge et al., 2018). Understanding that approach-avoidance tendencies fluctuate does, however, not imply that we can predict these fluctuations. This study was not designed to explain fluctuations in approach-avoidance tendencies^8^. Therefore, future studies should assess whether other variables can predict changes in approach-avoidance tendencies. For example, in our own lab, we found that food approach tendencies increase and decrease depending on BMI and hunger (Zech et al., unpublished manuscript).

In the current study, the order of AATs was not counterbalanced—the disgust AAT always followed the emotional expressions AAT. It is possible that the reliability of the disgust AAT was lower not because of the stimulus set, but because of order effects. Participants might have gotten tired or bored, for example, after completing the emotional expressions AAT, and temporal fluctuations in this effect could in turn have decreased reliability. An indication that this was the case, for example, is the overall reduced reaction times in the disgust AAT (although note that we still found the expected disgust avoidance tendency)^9^. Future studies comparing different stimulus sets in the AAT should therefore counterbalance the order of tasks to eliminate such order effects. It should also be mentioned that the error rate in the disgust AAT was overall higher than in the facial expression AAT. This difference could also explain differences in reliability, as error trials are removed before analysis, potentially leading to less stable approach-avoidance tendencies.

This study focused on a specific version of the AAT in which participants have to attend to the stimulus feature of interest because it has been shown that this design is more powerful than designs relying on more indirect instructions (Kersbergen et al., 2015; Lender et al., 2018; Meule, Richard, et al., 2019b; Phaf et al., 2014; Rotteveel & Phaf, 2004). However, this design also has the disadvantage of being less implicit, as it makes participants aware of the variable of interest (Rotteveel & Phaf, 2004). In certain cases, it might therefore be preferable to study approach-avoidance tendencies using a task variant in which participants are not instructed to attend to the stimulus feature of interest. Future studies should, therefore, directly compare the reliability of these two task designs.

Although we tested a large and diverse sample and included two distinct stimulus sets in this study, our results might still not be representative of other versions of the AAT and other stimulus sets. For example, in this experiment we used a smartphone-based version of the AAT and participants completed the task in a noncontrolled environment outside the laboratory. This environment might have increased measurement error (e.g., due to distractions) and in turn reduced the task reliability. In addition, our task used a different type of input (phone movement) than most other AATs (joystick movements). It is possible that this input may yield less or more reliable measurements than other AATs. To further the understanding of the AAT’s reliability, it is therefore advisable that researchers assess and report the reliability for their specific versions of the AAT and their specific stimulus sets to ensure reliable and reproduceable findings.

### Implications

Here, we demonstrated that the AAT’s test-retest reliability is insufficient to test individual differences or slow-changing variables. This finding matches those of earlier studies and findings from other implicit tasks (see Enkavi et al., 2019; Hedge et al., 2018) and implies that researchers who are interested in using the AAT as a measure of individual differences should use the task with care. Specifically, researchers should be aware that the task’s low test-retest reliability strongly limits their ability to correlate detected approach-avoidance tendencies with other individual difference variables (Spearman, 1904/2010)—potentially explaining failures to replicate prior findings. We, moreover, found that test-retest reliability did not depend on sample characteristics nor on the length of the retest period.

According to Hedge et al. (2018) poor test-retest reliability can be driven either by excessive measurement error, or by temporal fluctuations in the measured construct. To distinguish between these two possibilities, we also assessed the task’s split-half reliability. At least for the emotional expressions AAT, we showed that its split-half reliability is high enough for basic research (based on standards by Nunnally & Bernstein, 1994). This finding indicates that the AAT’s low test-retest reliability is more likely caused by temporal fluctuations in approach-avoidance tendencies (Hedge et al., 2018). This finding has both theoretical and practical implications:

Theoretically, our findings imply that approach-avoidance tendencies might be less stable than some would suggest. According to current theories of automaticity (Smith & DeCoster, 2000; Strack & Deutsch, 2004), behavioral tendencies are based on rigid memory systems that only change slowly, based on repeated exposure to new stimulus-response contingencies. Our findings imply that approach-avoidance tendencies can change relatively fast (over the period of one month). Theories explaining approach-avoidance tendencies should, therefore, be adapted to include such state-like changes. One way to integrate rigid memory systems and state-like changes in automatic behavior has been suggested by Gawronski and Bodenhausen (2006). According to these researchers, such dynamic changes in rigid automatic action tendencies could be explained through the principle of pattern activation (Smith, 1996). In this framework, automatic tendencies (e.g., approach tendencies) are not simply triggered by a stimulus (e.g., food), but by a combination of a context (e.g., hunger) with the stimulus. The principle of pattern activation thus allows for both slow-changing associative structures that drive approach-avoidance tendencies and state-like changes in these tendencies.

Practically, our findings imply that future AAT research—rather than focusing on using the task as a measure of individual differences—should aim at explaining why approach tendencies fluctuate over time. To our knowledge, few studies have focused on such state-like changes, possibly because traditional, computer-based versions of the AAT are stationary and difficult to use in longitudinal studies required to measure such changes (Zech et al., 2020). Modern, mobile AATs should facilitate such studies and could reveal predictors of temporal changes in future approach-avoidance studies (e.g., Meule et al., 2018; Zech et al., 2020). For example, in a recent study we showed that food approach tendencies increase when healthy-weight participants are hungry compared to satiated, with the opposite effect of hunger being present in overweight participants (Zech et al., unpublished manuscript).

Better understanding why approach-avoidance tendencies fluctuate should also help researchers who are interested in individual differences and slow-changing variables. Low reliability is at least partially driven by excessive unexplained variance (Liljequist et al., 2019). Studying and ultimately modeling fluctuations in approach-avoidance effects could therefore remove unexplained variance in individual difference studies, increasing reliability, observed correlations, and statistical power. For example, in a recent study we showed that the association between food approach tendencies and BMI only becomes apparent when hunger is also included in the model (Zech et al., 2021). Researchers interested in other types of approach-avoidance tendencies (e.g., towards addictive substances) should therefore also aim at better understanding why approach-avoidance tendencies fluctuate.

Understanding why approach-avoidance tendencies fluctuate is an incremental task which will likely take several years. In the meantime, the current findings point to some recommendations for researchers who want to use the AAT as an individual difference measure. Most importantly, researchers should not depend on single measurements, but rely on several measurements (for a similar conclusion in the context of internet interventions, see Schuster et al., 2020). In this study, we found that eight measurements increase the AATs reliability to moderate to good levels. It is possible that more measurements increase the AATs reliability even further. Smaller improvements can be achieved by carefully choosing stimulus sets and using recent methods of calculating approach-avoidance tendencies based on mixed models. Unlike double difference scores, mixed models do not compound error variance with systematic variance (Haines et al., 2020). In this study, we show that this too can somewhat improve the AATs reliability.

Finally, it should be noted that other methods have been successfully applied to increase the reliability of other cognitive tasks: for example, Waltmann et al. (2022) recently demonstrated that hierarchical modeling can improve the reliability of probabilistic reversal learning tasks; Skinner et al. (2018) demonstrated that increasing stimulus presentation times can improve the reliability of attentional bias measures; and Chevance et al. (2017) showed how less complex versions of the implicit association task (IAT) can increase the tasks’ reliability. Future AAT research could learn from these efforts to make the AAT even more reliable and further improve the task’s reproducibility.

### Conclusion

Although several researchers have already used the AAT as a measure of individual differences, its test-retest reliability has not yet been firmly established. Using a novel smartphone-based version of the AAT, this study established the task’s reliability in a large and diverse sample over a long retest period of up to eight months. We show that—when relying on single measurements sessions—the AAT’s test-retest reliability is too low to be used as a measure of individual differences. We further show that this low test-retest reliability is likely not driven by measurement error but by temporal fluctuations of approach-avoidance tendencies. Finally, our results reveal that multiple measures of the AAT are critical to increasing the test-retest reliability of the task. Because the AAT is the most widely used behavioral measure of approach and avoidance tendencies, our findings have broad implications for psychological assessment. Specifically, future AAT research should aim at better understanding what drives temporal fluctuations in approach-avoidance tendencies, for example, by using smartphone-based versions of the AAT that can be easily deployed in field research. Until the causes of these fluctuations are understood, researchers interested in the AAT as a trait measure should rely on multiple rather than single measurements in order to gain reliable and reproducible results.

## Figures and Tables

**Fig. 7:**
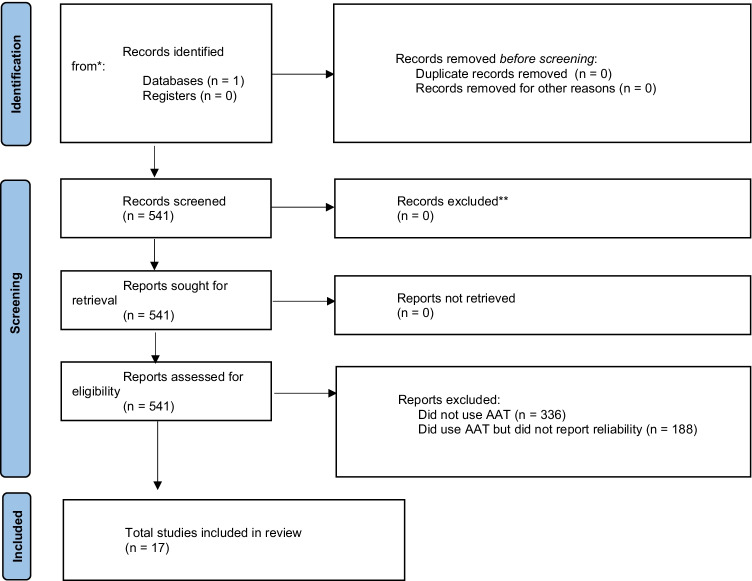
PRISMA flow diagram. This diagram gives an overview of the identification procedure of studies for this review.

## Data Availability

All data and materials used in this paper are openly available on the project’s Open Science Framework page (https://osf.io/t3f4y/)

## Appendix A: Comparison of studies

(Table 2)

**Table 2:** Comparison of studies

Study	N	Sample	AAT type	Stimuli	Instruction
Ernst et al. (2014)	42	alcohol-dependent/healthy control	joystick	alcohol/non-alcohol	relevant
Wiers et al. (2017)	45	alcohol-dependent/healthy control	joystick	alcohol/non-alcohol	irrelevant
Wiers et al. (2013)	64	smokers/abstinent smokers/nonsmokers	joystick	smoking/neutral	irrelevant
Machulska et al. (2015)	143	students	joystick	smoking/food	irrelevant
Maas et al. (2017)	94	students bothered by snacking habits/other habits	joystick	food/abstract pictures	irrelevant
Kakoschke et al. (2015)	146	female students	joystick	food/animals	irrelevant
Kakoschke et al. (2017a)	240	female students	joystick	food/animals	irrelevant
Kakoschke et al. (2017b)	245	female students	joystick	food/animals	irrelevant
Schumacher et al. (2016)	120	female students	joystick	chocolate/non-chocolate	irrelevant

## Appendix B: Systematic review

For this review, we followed the Preferred Reporting Items for Systematic Reviews and Meta-Analyses (PRISMA) guidelines (Moher et al., 2015). Relevant articles were identified through a search of one electronic database (PubMed) using the search term: *(("approach-avoidance task") OR ("approach-avoidance tendency") OR ("approach-avoidance bias") OR ("approach-avoidance conflict") OR ("approach tendency") OR ("approach bias"))*

This search yielded 541 articles. As it was not clear from the abstracts whether the articles reported reliability, we retrieved all articles. Of these 336 articles were excluded because they did not include any approach-avoidance task (AAT) studies. Of the included articles 188 did not report on the reliability of the AAT in measuring approach-avoidance tendencies. Of those that reported on the AAT’s reliability, four reported on the test-retest reliability of approach-avoidance tendencies.

(Figure 7)

## Appendix C: Split-half reliabilities

(Table 3)

**Table 3:** Overview of studies reporting split-half reliability

Study	N	Sample	AAT type	Stimuli	Instruction	Reliability
Baquedano et al. (2017)	50	meditators/non-meditators)	joystick	attractive/neutral foods	irrelevant	*r* = .51
Hahn et al. (2019)	102	students	joystick	alcohol/condoms	irrelevant	*r*s = .62, .33, .52
Kahveci et al. (2020)	60	female students	swipe	foods/objects	relevant	*r* = .58
Kahveci et al. (2021)	40	students	swipe	foods/objects	relevant	*rs* = .73–.76
Luo (2019, E2)	108	students	joystick	happy/angry faces	both	*α* = .49
Luo (2019, E3)	206	students	joystick	happy/angry faces	both	*α* = .71
Melkonyan et al. (2020)	37	men	joystick	high/low-craved foods	irrelevant	*r* = .55
Meule, Lender, et al. (2019a)	107	unselected	swipe	food/objects	relevant	*r* = .92
					irrelevant	*r*s = −.24; −.14
Rinck et al. (2018)	1405	abstinent alcohol-dependent	joystick	alcohol/non-alcohol	irrelevant	*r* = .58
Rinck et al. (2021)	86	students	swipe	spiders/butterflies	relevant	*r* = .13
Rodriguez-Nieto et al. (2019)	24	men	joystick (MRI)	sexual/neutral	relevant	*r* = .37
Schippers and Smid (2020)	149	male high-risk rapists/control	joystick	men/women	relevant	*r* = .97
Voegtle et al. (2021)	50	students	joystick	soft drinks/water	irrelevant	*r*s = .67; .24
Wen et al. (2020)	504	smokers	keyboard	cigarettes/neutral	irrelevant	*r* = .03
Wittekind et al. (2021)	45	students	joystick	chocolate/objects	relevant	*r =* .62
			swipe			*r =* .60
			mouse			*r =* .50
Zech (2020, E1)	55	female students	joystick	happy/angry faces	relevant	*r* = .8
			smartphone			*r =* .77
Zech (2020, E2)	151	unselected	smartphone	happy/angry faces	relevant	*r* = .91

This table shows split-half reliabilities for AAT studies identified in the literature review. *R*s refer to Spearman-Brown-corrected split-half reliabilities (except for the negative reliabilities of Meule, Lender, et al., 2019a, which are uncorrected, as the Spearman-Brown adjustment disproportionally inflates negative values. Alphas refer to Cronbach Alphas). The mean split-half reliability was .52 (*SD* = .30)

## Footnotes

1 Several researchers have suggested that differences in methodologies could explain different findings. However, the studies reviewed in this section used very similar versions of the AAT (see Appendix A; Table 2).

2 Note that three additional studies also examined the AAT’s reliability (Brown et al., 2014; Peeters et al., 2012; Reddy et al., 2016; see supplementary materials). However, these did not correct their approach tendencies using control stimuli and thus conflated approach-avoidance tendencies with main effects of movement direction (see Loijen et al., 2020; Zech et al., 2020). As these scores are not stimulus-specific and necessarily more reliable (for a comparison see for example Kahveci et al., 2020; Rinck et al., 2021), we do not consider them in this review, but include them in the supplementary materials.

3 Mixed models have several advantages over traditional ways of calculating approach-avoidance tendencies based on aggregation. First, mixed-models allow for greater flexibility in dealing with non-normally distributed data and inclusion of trial-level confounding variables (Hedge et al., 2018). Second, unlike aggregation methods, mixed models do not compound error variance with systematic variance: Aggregation methods follow a “two-stage summary approach” which assumes no measurement error in the first step. This in turn can lead to an attenuation of test-retest reliability (for a more complete explanation, see Haines et al., 2020).

4 We define “trait” here, as suggested by Polit (2014), as “an attribute with high temporal stability, such as intelligence” (2014).

5 Note that force can be measured as a secondary outcome variable using the mobile AAT (Zech et al., 2020). All force-related analyses can be found in the online materials.

6 Note that Shrout and Fleiss (1979) refer to these ICCs as multi-rater ICCs. Here, we use instead the name “average-measure ICCs,” suggested by Qin et al., 2019, as it is more intuitive in test–retest reliability studies.

7 Note that these recommendations were made for Cronbach's alpha coefficients. However, as split-half reliabilities from all possible splits converge to Cronbach’s alphas, we believe these recommendations are also applicable in our case.

8 Note, however, that in an exploratory analysis we were not able to predict fluctuations using two conceptually related variables taken from an overarching project (see supplementary materials).

9 Note that another explanation for the low reliability of the disgust AAT could be that overall higher measurement error could have caused lower test-retest reliabilities. However, even after correcting test-retest reliabilities for measurement error, the test-retest reliability of the disgust AAT was substantially lower than that of the emotional expressions AAT (see supplementary materials).

## Code Availability

The source code of the mobile AAT used to collect data in this study is openly available on the project’s Open Science Framework page (https://osf.io/t3f4y/).
